# Crystal structure of 1-[(2,2-dimethyl-1,3-dioxolan-4-yl)meth­yl]-2-(thia­zol-4-yl)-1*H*-benzimidazole

**DOI:** 10.1107/S205698901502085X

**Published:** 2015-11-18

**Authors:** Hicham Gueddar, Rachid Bouhfid, El Mokhtar Essassi, Mohamed Saadi, Lahcen El Ammari

**Affiliations:** a Moroccan Foundation for Advanced Science, Innovation and Research (MASCIR), Rabat, Morocco; b Laboratoire de Chimie Organique Hétérocyclique URAC 21, Pôle de Compétence Pharmacochimie, Av. Ibn Battouta, BP 1014, Faculté des Sciences, Université Mohammed V de Rabat, Morocco; c Laboratoire de Chimie du Solide Appliquée, Faculté des Sciences, Université Mohammed V de Rabat, Avenue Ibn Battouta, BP 1014, Rabat, Morocco

**Keywords:** crystal structure, benzimidazole, thia­zol-4-yl, 1,3-dioxolan-4-yl

## Abstract

The benzimidazole ring in the title compound, C_16_H_17_N_3_O_2_S, is almost planar, with the greatest deviation from the mean plane being 0.032 (1) Å. The fused-ring system makes dihedral angles of 19.91 (7) and 24.51 (8)° with the best plane through each of the thia­zol-4-yl and 1,3-dioxolan-4-yl rings, respectively; the latter exhibits an envelope conformation with the methyl­ene C atom being the flap. Finally, the thia­zol-4-yl ring makes a dihedral angle of 33.85 (9)° with the 1,3-dioxolan-4-yl ring. In the crystal, mol­ecules are connected by a pair of C—H⋯π(imidazole) inter­actions to form centrosymmetric aggregates.

## Related literature   

For the use of the title compound as an anthelmintic, see: Brown *et al.* (1961 ▸); Hennekeuser *et al.* (1969 ▸); as a food preservative and an agricultural fungicide, see: Arenas & Johnson (1994 ▸); for induction of aneuploidy and photogenotoxicity in bacteria and cultured human cells, see: Watanabe-Akanuma *et al.* (2005 ▸); as an anti-angiogenic, see: Cha *et al.* (2012 ▸); and as a ligand for transition metal ions, see: Gueddar *et al.* (2013 ▸).
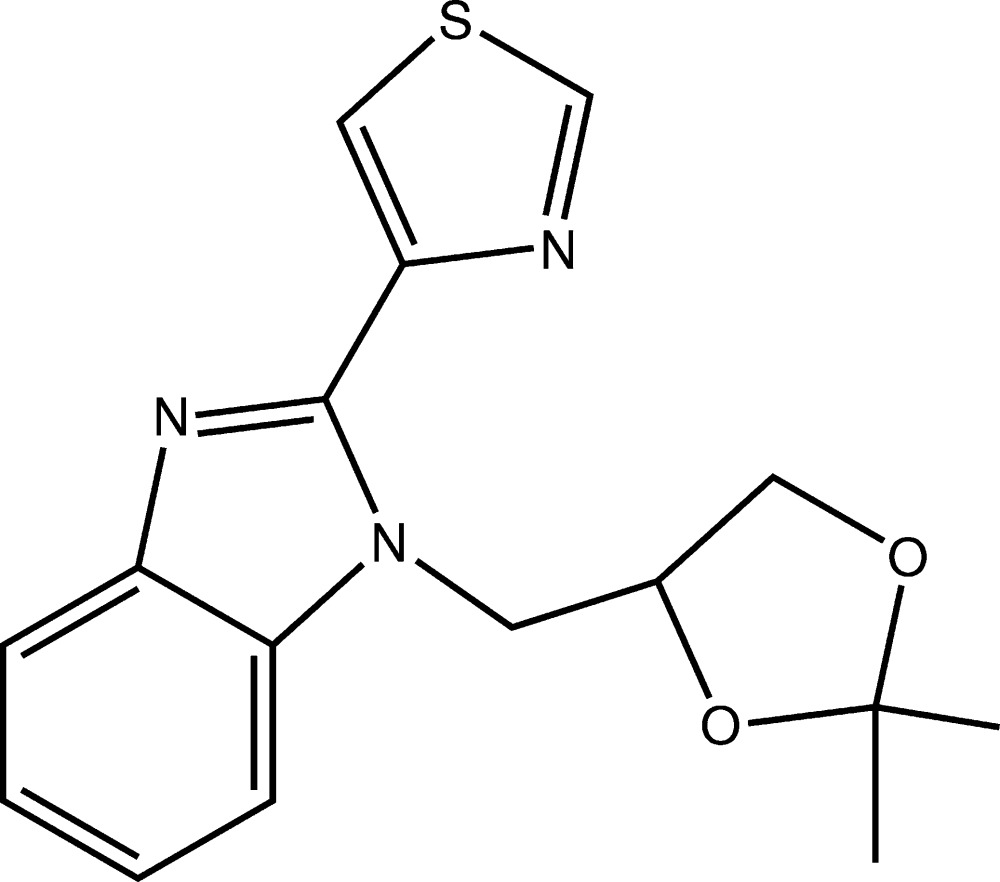


## Experimental   

### Crystal data  

C_16_H_17_N_3_O_2_S
*M* *_r_* = 315.38
Triclinic, 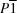

*a* = 9.3177 (5) Å
*b* = 9.3786 (6) Å
*c* = 9.5418 (6) Å
α = 78.739 (4)°
β = 78.777 (3)°
γ = 73.632 (3)°
*V* = 775.95 (8) Å^3^
*Z* = 2
Mo *K*α radiation
μ = 0.22 mm^−1^
*T* = 296 K
0.36 × 0.31 × 0.26 mm

### Data collection  

Bruker X8 APEX diffractometer
Absorption correction: multi-scan (*SADABS*; Bruker, 2009 ▸) *T*_min_ = 0.700, *T*_max_ = 0.747
19810 measured reflections
4740 independent reflections
2804 reflections with *I* > 2σ(*I*)
*R*_int_ = 0.037

### Refinement  

*R*[*F* ^2^ > 2σ(*F* ^2^)] = 0.043
*wR*(*F* ^2^) = 0.116
*S* = 1.03
4740 reflections
199 parameters
H-atom parameters constrained
Δρ_max_ = 0.16 e Å^−3^
Δρ_min_ = −0.32 e Å^−3^

### 

Data collection: *APEX2* (Bruker, 2009 ▸); cell refinement: *SAINT-Plus* (Bruker, 2009 ▸); data reduction: *SAINT-Plus*; program(s) used to solve structure: *SHELXS2014*/ (Sheldrick, 2008 ▸); program(s) used to refine structure: *SHELXL2014* (Sheldrick, 2015 ▸); molecular graphics: *ORTEP-3 for Windows* (Farrugia, 2012 ▸); software used to prepare material for publication: *PLATON* (Spek, 2009 ▸) and *publCIF* (Westrip,2010 ▸).

## Supplementary Material

Crystal structure: contains datablock(s) I. DOI: 10.1107/S205698901502085X/tk5404sup1.cif

Structure factors: contains datablock(s) I. DOI: 10.1107/S205698901502085X/tk5404Isup2.hkl

Supporting information file. DOI: 10.1107/S205698901502085X/tk5404Isup3.cml

**Fig. 1.**
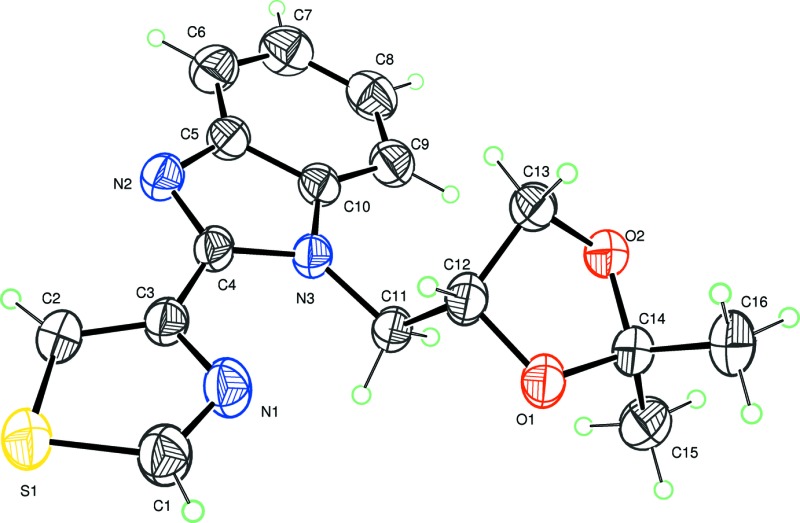
Molecular structure of the title compound with the atom-labelling scheme. Displacement ellipsoids are drawn at the 50% probability level. H atoms are represented as small circles.

. DOI: 10.1107/S205698901502085X/tk5404fig1.tif
Mol­ecular structure of the title compound with the atom-labelling scheme. Displacement ellipsoids are drawn at the 50% probability level. H atoms are represented as small circles.

CCDC reference: 1435046

Additional supporting information:  crystallographic information; 3D view; checkCIF report

## Figures and Tables

**Table 1:** Hydrogen-bond geometry (Å, °) *Cg*1 is the centroid of the N2/N3/C4/C5/C10 ring.

*D*—H⋯*A*	*D*—H	H⋯*A*	*D*⋯*A*	*D*—H⋯*A*
C13—H13*B*⋯*Cg*1^i^	0.97	2.83	3.7543 (18)	160

Symmetry code: (i) 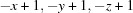.

## supplementary crystallographic information

### S1. Comment

Thiabendazole [2-(4-thiazolyl)benzimidazole, TBZ], is used as a broad spectrum anthelmintic in various animals (Brown *et al.*, 1961) and in humans (Hennekeuser *et al.*, 1969). TBZ inhibits anaerobic respiration at the level of mitochondrial helminth-specific enzyme. This compound has also been used as a food preservative and an agricultural fungicide (Arenas and Johnson, 1994). Induction of aneuploidy and photogenotoxicity has been reported for TBZ in bacteria and cultured human cells (Watanabe-Akanuma *et al.*, 2005). TBZ has recently been verified to be vascular disrupting agent and thus as a potential complementary therapeutic for use in combination with current anti-angiogenic therapeutics (Cha *et al.*, 2012). TBZ is also an effective ligand to coordinate transition metal ions (Gueddar *et al.*, 2013). The title compound is synthesized by action of TBZ on tosylated solketal under phase transfer catalysis conditions.

The molecule of the title compound is build up from fused five- and six-membered rings linked to a thiazol-4-yl cycle and, *via* one —CH_2_— link, to a 2,2-dimethyl-1,3-dioxolan-4-yl group as shown in Fig. 1. The benzimidazole ring is essentially planar with the maximum deviation from the mean plane being 0.032 (1)° at C4 and makes dihedral angles of 19.91 (7) and 24.51 (8)° with the mean plane through the thiazol-4-yl and the 3-dioxolan-4-yl rings, respectively. The dihedral angle between the thiazol-4-yl cycle and the 3-dioxolan-4-yl ring is of 33.85 (9)°. Furthermore, the five-membered ring (O1O2C12C13C14) adopts an envelope conformations on C13 as indicated by the total puckering amplitude Q2 = 0.381 (2) Å and spherical polar angle φ2 = 72.2 (2)°. In the crystal, the molecules are held together by C13–H13B···π interactions involving the imidazole ring.

### S2. Experimental

To a solution of thiabendazole (1 g, 5 × 10 ^-3^ mol.) dissolved in DMF (20 ml) was added potassium carbonate (0.83 g, 6 × 10 ^-3^ mol.), tetra-*n*-butylammonium bromide (0.13 g, 0.4 × 10 ^-3^ mol) and tosylated solketal (2.79 g, 10 × 10 ^-3^ mol). The mixture was heated for 48 h. After the completion of the reaction (as monitored by TLC), the inorganic material was filtered and the solvent was removed under reduced pressure. The residue obtained was recrystallized from ethanol to afford the title compound as colourless crystals.

### S3. Refinement

The H atoms were located in a difference map and treated as riding with C—H = 0.93 Å (aromatic), C—H = 0.97 (methylene), C—H = 0.98 (methine) and C—H = 0.96 Å (methyl), and with *U*_iso_(H) = 1.2 *U*_eq_(aromatic, methine and methylene) and *U*_iso_(H) = 1.5 *U*_eq_(methyl). The reflections (0 1 0) and (1 0 0), which were affected by the beam-stop, were removed from the final cycles of refinement owing to poor agreement.

### Crystal data

**Table:**

C_16_H_17_N_3_O_2_S	*Z* = 2
*M**_r_* = 315.38	*F*(000) = 332
Triclinic, *P*1	*D*_x_ = 1.350 Mg m^−^^3^
*a* = 9.3177 (5) Å	Mo *K*α radiation, λ = 0.71073 Å
*b* = 9.3786 (6) Å	Cell parameters from 4740 reflections
*c* = 9.5418 (6) Å	θ = 2.8–30.5°
α = 78.739 (4)°	µ = 0.22 mm^−^^1^
β = 78.777 (3)°	*T* = 296 K
γ = 73.632 (3)°	Block, colourless
*V* = 775.95 (8) Å^3^	0.36 × 0.31 × 0.26 mm

### Data collection

**Table:**

Bruker X8 APEX diffractometer	4740 independent reflections
Radiation source: fine-focus sealed tube	2804 reflections with *I* > 2σ(*I*)
Graphite monochromator	*R*_int_ = 0.037
φ and ω scans	θ_max_ = 30.5°, θ_min_ = 2.8°
Absorption correction: multi-scan (*SADABS*; Bruker, 2009)	*h* = −13→13
*T*_min_ = 0.700, *T*_max_ = 0.747	*k* = −13→13
19810 measured reflections	*l* = −13→13

### Refinement

**Table:**

Refinement on *F*^2^	0 restraints
Least-squares matrix: full	Hydrogen site location: inferred from neighbouring sites
*R*[*F*^2^ > 2σ(*F*^2^)] = 0.043	H-atom parameters constrained
*wR*(*F*^2^) = 0.116	*w* = 1/[σ^2^(*F*_o_^2^) + (0.0449*P*)^2^ + 0.0871*P*] where *P* = (*F*_o_^2^ + 2*F*_c_^2^)/3
*S* = 1.03	(Δ/σ)_max_ < 0.001
4740 reflections	Δρ_max_ = 0.16 e Å^−^^3^
199 parameters	Δρ_min_ = −0.32 e Å^−^^3^

### Special details

**Table:**

Geometry. All e.s.d.'s (except the e.s.d. in the dihedral angle between two l.s. planes) are estimated using the full covariance matrix. The cell e.s.d.'s are taken into account individually in the estimation of e.s.d.'s in distances, angles and torsion angles; correlations between e.s.d.'s in cell parameters are only used when they are defined by crystal symmetry. An approximate (isotropic) treatment of cell e.s.d.'s is used for estimating e.s.d.'s involving l.s. planes.

### Fractional atomic coordinates and isotropic or equivalent isotropic displacement parameters (Å^2^)

**Table:**

	*x*	*y*	*z*	*U*_iso_*/*U*_eq_	
C1	0.9210 (2)	−0.0317 (2)	0.68651 (19)	0.0621 (5)	
H1	1.0193	−0.0386	0.6391	0.074*	
C2	0.69556 (19)	−0.05253 (19)	0.84926 (17)	0.0567 (4)	
H2	0.6188	−0.0714	0.9230	0.068*	
C3	0.67345 (17)	0.04071 (16)	0.72279 (15)	0.0455 (3)	
C4	0.52432 (17)	0.11882 (16)	0.68306 (15)	0.0431 (3)	
C5	0.28330 (18)	0.20058 (17)	0.69744 (17)	0.0480 (4)	
C6	0.12767 (19)	0.2376 (2)	0.7404 (2)	0.0625 (5)	
H6	0.0870	0.2227	0.8375	0.075*	
C7	0.0354 (2)	0.2967 (2)	0.6362 (2)	0.0700 (5)	
H7	−0.0690	0.3218	0.6636	0.084*	
C8	0.0949 (2)	0.3197 (2)	0.4904 (2)	0.0672 (5)	
H8	0.0292	0.3598	0.4225	0.081*	
C9	0.2485 (2)	0.28454 (19)	0.44413 (19)	0.0569 (4)	
H9	0.2883	0.2997	0.3467	0.068*	
C10	0.34127 (17)	0.22519 (16)	0.55071 (16)	0.0450 (3)	
C11	0.60240 (18)	0.16781 (16)	0.40757 (15)	0.0454 (3)	
H11A	0.5501	0.1612	0.3315	0.055*	
H11B	0.6843	0.0776	0.4179	0.055*	
C12	0.66868 (18)	0.30373 (17)	0.36290 (16)	0.0477 (4)	
H12	0.7195	0.3150	0.4394	0.057*	
C13	0.55626 (19)	0.44806 (18)	0.31814 (17)	0.0535 (4)	
H13A	0.4595	0.4562	0.3802	0.064*	
H13B	0.5932	0.5348	0.3188	0.064*	
C14	0.69603 (17)	0.36581 (17)	0.11179 (16)	0.0488 (4)	
C15	0.6876 (2)	0.2593 (2)	0.01678 (19)	0.0673 (5)	
H15A	0.7877	0.2135	−0.0270	0.101*	
H15B	0.6277	0.3134	−0.0572	0.101*	
H15C	0.6420	0.1827	0.0738	0.101*	
C16	0.7744 (2)	0.4856 (2)	0.0327 (2)	0.0702 (5)	
H16A	0.8749	0.4389	−0.0094	0.105*	
H16B	0.7793	0.5480	0.0993	0.105*	
H16C	0.7187	0.5459	−0.0422	0.105*	
N1	0.80427 (15)	0.05273 (16)	0.62983 (15)	0.0575 (4)	
N2	0.40020 (14)	0.13467 (15)	0.77840 (13)	0.0487 (3)	
N3	0.49717 (14)	0.17320 (13)	0.54286 (12)	0.0428 (3)	
O1	0.77265 (12)	0.28446 (12)	0.23223 (11)	0.0532 (3)	
O2	0.54583 (12)	0.43257 (12)	0.17557 (11)	0.0528 (3)	
S1	0.88238 (5)	−0.12939 (6)	0.85352 (5)	0.06612 (16)	

### Atomic displacement parameters (Å^2^)

**Table:**

	*U*^11^	*U*^22^	*U*^33^	*U*^12^	*U*^13^	*U*^23^
C1	0.0568 (10)	0.0661 (12)	0.0570 (10)	−0.0168 (9)	−0.0043 (8)	0.0032 (9)
C2	0.0634 (10)	0.0589 (10)	0.0394 (8)	−0.0103 (8)	−0.0008 (7)	−0.0019 (7)
C3	0.0575 (9)	0.0398 (8)	0.0378 (8)	−0.0142 (7)	−0.0019 (7)	−0.0050 (6)
C4	0.0582 (9)	0.0353 (7)	0.0353 (7)	−0.0151 (6)	−0.0030 (6)	−0.0028 (6)
C5	0.0571 (9)	0.0408 (8)	0.0460 (8)	−0.0155 (7)	−0.0027 (7)	−0.0067 (7)
C6	0.0574 (10)	0.0646 (11)	0.0609 (11)	−0.0161 (8)	0.0029 (8)	−0.0092 (9)
C7	0.0560 (10)	0.0694 (13)	0.0828 (14)	−0.0143 (9)	−0.0089 (10)	−0.0110 (11)
C8	0.0697 (12)	0.0584 (11)	0.0753 (13)	−0.0103 (9)	−0.0268 (10)	−0.0070 (9)
C9	0.0713 (11)	0.0475 (9)	0.0522 (10)	−0.0137 (8)	−0.0138 (8)	−0.0055 (7)
C10	0.0560 (9)	0.0344 (7)	0.0455 (8)	−0.0143 (6)	−0.0063 (7)	−0.0053 (6)
C11	0.0600 (9)	0.0392 (8)	0.0350 (7)	−0.0138 (7)	−0.0013 (6)	−0.0045 (6)
C12	0.0630 (9)	0.0460 (9)	0.0358 (7)	−0.0202 (7)	−0.0048 (7)	−0.0032 (6)
C13	0.0705 (10)	0.0408 (8)	0.0465 (9)	−0.0163 (8)	0.0016 (8)	−0.0071 (7)
C14	0.0540 (9)	0.0481 (9)	0.0383 (8)	−0.0113 (7)	−0.0030 (7)	0.0013 (7)
C15	0.0823 (13)	0.0676 (12)	0.0505 (10)	−0.0155 (10)	−0.0057 (9)	−0.0144 (9)
C16	0.0817 (13)	0.0655 (12)	0.0573 (11)	−0.0289 (10)	0.0011 (9)	0.0091 (9)
N1	0.0523 (8)	0.0596 (9)	0.0537 (8)	−0.0172 (7)	−0.0055 (6)	0.0095 (7)
N2	0.0546 (7)	0.0489 (7)	0.0405 (7)	−0.0155 (6)	−0.0004 (6)	−0.0048 (6)
N3	0.0534 (7)	0.0380 (7)	0.0354 (6)	−0.0131 (5)	−0.0029 (5)	−0.0029 (5)
O1	0.0508 (6)	0.0579 (7)	0.0422 (6)	−0.0102 (5)	−0.0041 (5)	0.0049 (5)
O2	0.0562 (6)	0.0514 (6)	0.0429 (6)	−0.0066 (5)	−0.0048 (5)	−0.0014 (5)
S1	0.0670 (3)	0.0732 (3)	0.0465 (3)	−0.0051 (2)	−0.0116 (2)	0.0032 (2)

### Geometric parameters (Å, º)

**Table:**

C1—N1	1.295 (2)	C10—N3	1.3879 (19)
C1—S1	1.7030 (18)	C11—N3	1.4612 (18)
C1—H1	0.9300	C11—C12	1.522 (2)
C2—C3	1.359 (2)	C11—H11A	0.9700
C2—S1	1.6913 (18)	C11—H11B	0.9700
C2—H2	0.9300	C12—O1	1.4300 (17)
C3—N1	1.3794 (19)	C12—C13	1.504 (2)
C3—C4	1.461 (2)	C12—H12	0.9800
C4—N2	1.3167 (18)	C13—O2	1.4205 (19)
C4—N3	1.3807 (18)	C13—H13A	0.9700
C5—N2	1.388 (2)	C13—H13B	0.9700
C5—C6	1.390 (2)	C14—O2	1.4299 (18)
C5—C10	1.398 (2)	C14—O1	1.4431 (18)
C6—C7	1.372 (3)	C14—C15	1.501 (2)
C6—H6	0.9300	C14—C16	1.510 (2)
C7—C8	1.393 (3)	C15—H15A	0.9600
C7—H7	0.9300	C15—H15B	0.9600
C8—C9	1.378 (3)	C15—H15C	0.9600
C8—H8	0.9300	C16—H16A	0.9600
C9—C10	1.393 (2)	C16—H16B	0.9600
C9—H9	0.9300	C16—H16C	0.9600
			
N1—C1—S1	115.57 (13)	O1—C12—C11	108.49 (12)
N1—C1—H1	122.2	C13—C12—C11	114.15 (13)
S1—C1—H1	122.2	O1—C12—H12	110.6
C3—C2—S1	110.39 (12)	C13—C12—H12	110.6
C3—C2—H2	124.8	C11—C12—H12	110.6
S1—C2—H2	124.8	O2—C13—C12	101.61 (12)
C2—C3—N1	114.69 (14)	O2—C13—H13A	111.4
C2—C3—C4	123.77 (14)	C12—C13—H13A	111.4
N1—C3—C4	121.50 (13)	O2—C13—H13B	111.4
N2—C4—N3	113.24 (13)	C12—C13—H13B	111.4
N2—C4—C3	122.47 (13)	H13A—C13—H13B	109.3
N3—C4—C3	124.13 (13)	O2—C14—O1	104.86 (11)
N2—C5—C6	130.26 (15)	O2—C14—C15	108.45 (13)
N2—C5—C10	110.23 (13)	O1—C14—C15	110.33 (13)
C6—C5—C10	119.47 (16)	O2—C14—C16	110.57 (13)
C7—C6—C5	118.54 (17)	O1—C14—C16	109.05 (13)
C7—C6—H6	120.7	C15—C14—C16	113.24 (15)
C5—C6—H6	120.7	C14—C15—H15A	109.5
C6—C7—C8	121.29 (17)	C14—C15—H15B	109.5
C6—C7—H7	119.4	H15A—C15—H15B	109.5
C8—C7—H7	119.4	C14—C15—H15C	109.5
C9—C8—C7	121.69 (18)	H15A—C15—H15C	109.5
C9—C8—H8	119.2	H15B—C15—H15C	109.5
C7—C8—H8	119.2	C14—C16—H16A	109.5
C8—C9—C10	116.57 (17)	C14—C16—H16B	109.5
C8—C9—H9	121.7	H16A—C16—H16B	109.5
C10—C9—H9	121.7	C14—C16—H16C	109.5
N3—C10—C9	131.86 (14)	H16A—C16—H16C	109.5
N3—C10—C5	105.65 (13)	H16B—C16—H16C	109.5
C9—C10—C5	122.43 (15)	C1—N1—C3	109.91 (14)
N3—C11—C12	113.34 (12)	C4—N2—C5	104.91 (12)
N3—C11—H11A	108.9	C4—N3—C10	105.96 (12)
C12—C11—H11A	108.9	C4—N3—C11	129.77 (13)
N3—C11—H11B	108.9	C10—N3—C11	124.02 (12)
C12—C11—H11B	108.9	C12—O1—C14	108.64 (11)
H11A—C11—H11B	107.7	C13—O2—C14	106.22 (12)
O1—C12—C13	101.94 (12)	C2—S1—C1	89.43 (8)

### Hydrogen-bond geometry (Å, º)

Cg1 is the centroid of the N2/N3/C4/C5/C10 ring.

**Table:**

*D*—H···*A*	*D*—H	H···*A*	*D*···*A*	*D*—H···*A*
C13—H13*B*···*Cg*1^i^	0.97	2.83	3.7543 (18)	160

Symmetry code: (i) −*x*+1, −*y*+1, −*z*+1.
