# Supplementary figures and images for: Crystal structure of 1-[(2,2-dimethyl-1,3-dioxolan-4-yl)meth­yl]-2-(thia­zol-4-yl)-1H-benzimidazole
Source: Acta Crystallogr E Crystallogr Commun. 2015 Nov 18;71(Pt 12):o951–2. doi: 10.1107/S205698901502085X (PMC4719918; doi:10.1107/S205698901502085X)

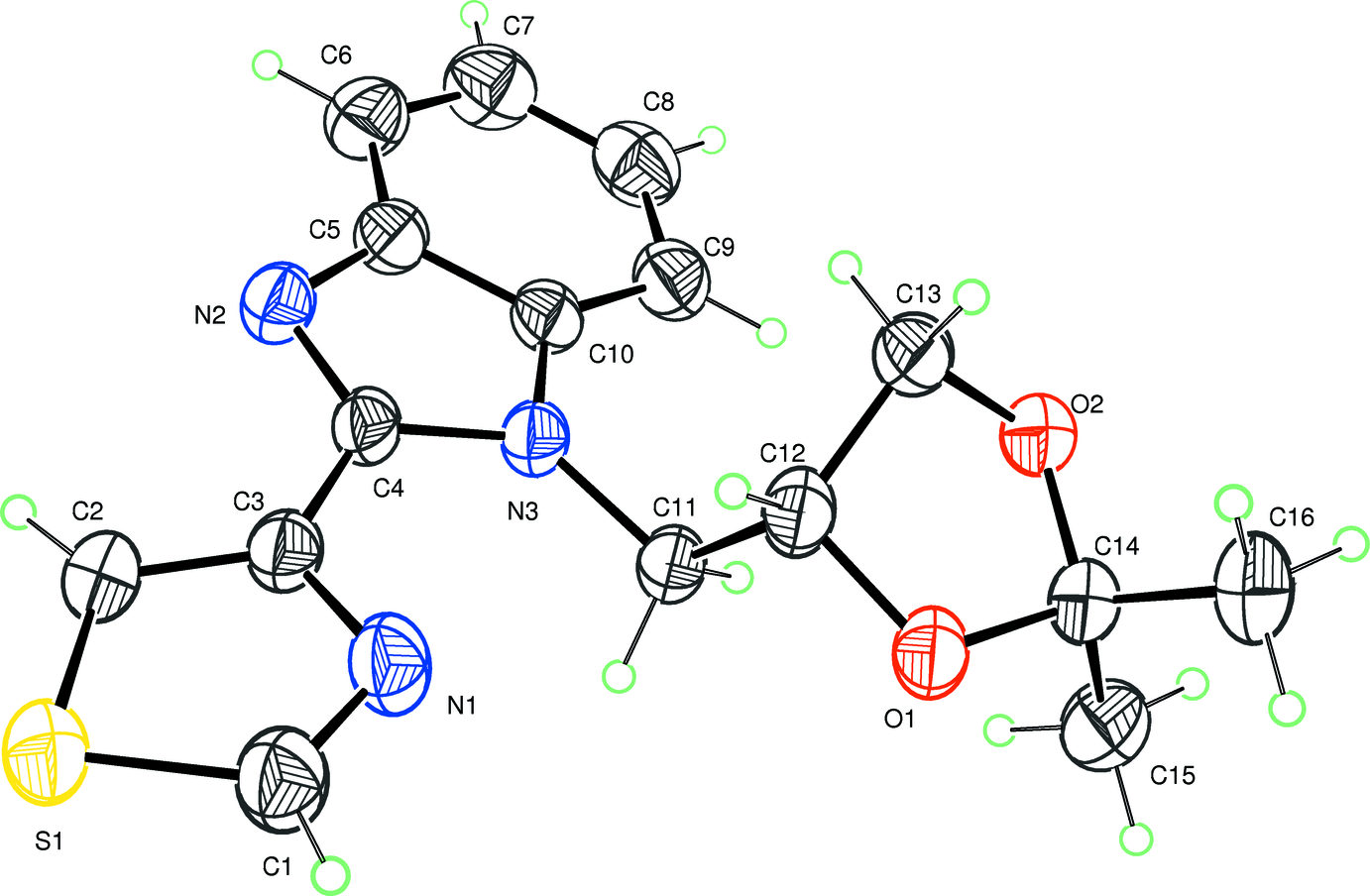

Supplement: Supplementary file 4 [file e-71-0o951-fig1.tif]
